# Selenium Bioaccumulation in *Sanghuangporus sanghuang*: Source-Specific Regulation of Fruiting Body Development, Selenium Speciation, and Nutritional Quality

**DOI:** 10.3390/foods15091575

**Published:** 2026-05-03

**Authors:** Taizeng Xin, Meina He, Tengye Luan, Ning Jiang, Feng Zhou, Lei Zha, Xiaodong Shang, Haoran Dong, Hailong Yu

**Affiliations:** 1 Engineering Research Centre of Chinese Ministry of Education for Edible and Medicinal Fungi, Jilin Agricultural University, Changchun 130118, China; xintaizeng0111@163.com (T.X.); luantengye01@163.com (T.L.); 2 Institute of Edible Fungi, Shanghai Academy of Agricultural Sciences, National Engineering Research Center of Edible Fungi, Shanghai 201403, China; 20240506@saas.sh.cn (M.H.); jiangning@saas.sh.cn (N.J.); feng5412@126.com (F.Z.); zhalei@saas.sh.cn (L.Z.); xdshang@163.com (X.S.)

**Keywords:** *Sanghuangporus sanghuang*, selenium source, fruiting body development, selenium speciation, nutritional quality, standardized cultivation

## Abstract

*Sanghuangporus sanghuang* (*S. sanghuang*) is an important medicinal mushroom rich in bioactive compounds. Selenium (Se) biofortification may further enhance its functional value and industrial profitability; however, evidence-based guidance on Se source selection and dosage for production remains insufficient. Using the strain “*Sanghuang* Hu2”, we compared sodium selenite, nano-selenium (nano-Se), and selenium-enriched yeast (Se-yeast) at different supplementation levels and comprehensively evaluated their effects on mycelial growth and fruiting body development, Se accumulation and speciation, and nutritional quality. The responses of *S. sanghuang* were strongly Se-source-specific and concentration-dependent. Se-yeast caused the least inhibition of mycelial growth while achieving the highest Se uptake and biotransformation efficiency. During bag cultivation, supplementation with 15 mg/kg Se-yeast significantly increased single-bag yield and biological efficiency without prolonging full colonization time and exhibited superior input cost performance. This treatment enabled an extremely high proportion of organic Se accumulation (>99.5%), dominated by selenomethionine. Moreover, Se-yeast markedly improved crude protein, crude polysaccharides, and total amino acids in fruiting bodies, with lysine showing the largest increase. Overall, considering growth and yield, Se accumulation/speciation, nutritional enhancement, and economic feasibility, Se-yeast is the optimal Se source for Se-enriched *Sanghuang*, with a recommended dosage of 15 mg/kg.

## 1. Introduction

*Sanghuangporus sanghuang* (*S. sanghuang*) is a traditional Chinese medicinal mushroom with a long history of use [1]. Modern studies have shown that *Sanghuangporus* species, including *S. sanghuang*, are rich in bioactive constituents such as polysaccharides, polyphenols, flavonoids, and triterpenoids and exhibit diverse biological activities, including antioxidant, immunomodulatory, hypoglycemic, and antitumor effects [2,3,4,5]. With the expanding market for functional agricultural products, nutritional fortification has become an important strategy to enhance the value chain of *S. sanghuang* and promote industrial upgrading.

Selenium is an essential trace element for humans and participates in the synthesis and metabolism of important selenoproteins and antioxidant enzymes, such as glutathione peroxidase, thereby contributing to redox regulation, immune function, and health maintenance [6,7,8]. In China, average daily Se intake commonly fails to meet the recommended 50 μg/d specified by the health industry standard (WS/T 578.3-2017) [9]. Dietary Se supplementation is a safe and effective approach. Exogenous Se addition to cultivation substrates can increase Se content in agricultural products, thereby improving Se intake through diet. Due to their short growth cycle, high Se biotransformation efficiency, and strong adaptability, edible mushrooms are considered ideal carriers for Se biofortification [10]. Se-enriched mushrooms retain their baseline nutritional value while converting inorganic Se into safer and more functional organic forms, thereby integrating the dual value of “food–medicine homology” and “trace-element fortification” [11].

Current research on Se-enriched edible fungi has focused mainly on widely cultivated species such as *Lentinula edodes*, *Pleurotus* spp., *Flammulina velutipes*, and *Agaricus bisporus* [12,13,14,15,16,17], whereas systematic studies on *S. sanghuang*, a high-value medicinal mushroom, remain limited [18,19]. Previous reports indicated that appropriate Se supplementation can significantly improve yield, Se accumulation, and nutritional quality of mushrooms [20,21,22,23]. Sodium selenite, the most widely used inorganic Se source, enables efficient enrichment but may induce toxicity at high doses [11]. In contrast, organic Se sources may better support mycelial metabolism, while the bioavailability and application performance of nano-Se in mushrooms require further validation. Therefore, screening suitable Se sources and optimal dosages for *S. sanghuang* is of substantial practical importance for standardized Se-enriched cultivation.

Accordingly, this study used the *S. sanghuang* strain “*Sanghuang* Hu2” to systematically compare sodium selenite, nano-Se, and Se-yeast at different concentrations in terms of mycelial growth, fruiting body development, Se accumulation characteristics, and nutritional quality. The objectives were to identify the optimal Se source and dosage, elucidate regulatory patterns of Se on growth and quality formation, and provide theoretical and technical support for standardized Se-enriched *Sanghuang* production.

## 2. Materials and Methods

### 2.1. Strain and Media

The *S. sanghuang* strain “*Sanghuang* Hu2” was provided by the National Edible Fungi Germplasm Resource Bank (Shanghai, China).

Potato dextrose agar (PDA) medium was prepared using potato decoction from 200 g potatoes, glucose 20 g, and agar 20 g, with distilled water added to a final volume of 1000 mL.

The cultivation substrate consisted of sawdust 70%, wheat bran 20%, corn flour 9%, and CaCO_3_ 1% (all on a dry-weight basis, *w*/*w*). Distilled water was added to adjust the final substrate moisture content to 60–65%.

### 2.2. Reagents and Major Instruments

Se-yeast was purchased from Shanghai Yuanye Bio-Technology Co., Ltd. (Shanghai, China). Sodium selenite was obtained from Sinopharm Chemical Reagent Co., Ltd. (Shanghai, China). Nano-selenium was purchased from Hubei Baiteke Bioengineering Co., Ltd. (Wuhan, China; catalogue No. 6971124230021), with a particle size range of 10–100 nm according to the manufacturer’s specifications.

The main instruments used in this study included an electronic balance (HTP-312, Huachao Industrial Co., Ltd., Shanghai, China), a constant-temperature incubator (ZXSD-B1430, Zhicheng Analytical Instrument Manufacturing Co., Ltd., Shanghai, China), an electric thermostatic drying oven (DHG-9620A, Yiheng Scientific Instrument Co., Ltd., Shanghai, China), and an atomic fluorescence spectrometer (AFS-822, Beijing Jitian Instrument Co., Ltd., Beijing, China).

### 2.3. Experimental Design

#### 2.3.1. PDA Plate Cultivation of Mycelia

Cold-stored cultures (4 °C) were reactivated and grown on PDA slants at 25 °C until fully covered. Uniform mycelial plugs (5 mm diameter) were excised and transferred to the center of PDA plates containing different Se sources and concentrations. Three Se sources were tested, each at four concentration gradients (1, 5, 10, 15 mg/L). Plates without Se served as the control (CK). Each treatment had five replicates. Plates were incubated at 25 °C in darkness (inverted). Colony diameters were measured on day 2 (T1) and day 5 (T2) using the cross method (two measurements each time, D1 and D2). Mycelial density, color, and colony morphology were recorded.

Mycelial growth rate was calculated as:

(1) $$S=\frac{D2-D1}{(T2-T1)\times 2}$$

where S is growth rate (mm/d); D1 and D2 are colony diameters (mm) at T1 and T2 (d), respectively.

#### 2.3.2. Bag Cultivation of Fruiting Bodies

Before sterilization, Se sources were added to the cultivation substrate to obtain Se concentrations of 0 (CK), 15, and 70 mg/kg. For each treatment, 100 cultivation bags were prepared (≈500 g dry substrate per bag). Bags were sterilized at 121 °C for 2 h, cooled to room temperature, and inoculated aseptically. During spawn run, bags were incubated at 25 °C in darkness. When mycelia reached the “bag shoulder”, the mycelial front was marked and the time to full colonization was recorded. After colonization, bags were removed and fruiting was induced by spraying for humidity (RH 85–90%) and light regulation (1000–1500 lx, 12 h/d). Fruiting bodies were harvested at 115–120 d when the pileus margin changed from pale yellow to golden yellow and became tough, following the collection standard [24]. Fruiting bodies were dried at 55 °C to constant weight, pulverized, and passed through an 80-mesh sieve for subsequent analyses.

### 2.4. Measurement Indices and Methods

#### 2.4.1. Selenium Content Determination

Wet digestion was selected for selenium quantification in dried samples according to the national standard GB 5009.93-2017. This method showed slightly better analytical performance than microwave-assisted digestion, including better precision (RSD: 0.35% vs. 0.52% for the tea reference material GBW 10016, *n* = 8) and slightly higher spiked recovery (94% vs. 92%). It also offered lower equipment cost and higher sample throughput for routine batch analysis. Since both methods rely on mixed-acid oxidation of organic matter, the use of wet digestion under standardized national operating specifications was considered appropriate for selenium determination in this study.

Total Se was determined according to GB 5009.93-2017 (Atomic Fluorescence Spectrometry) [25]. Briefly, 0.2 g dried sample was digested by wet digestion using mixed nitric/perchloric acid, brought to volume, reduced with hydrochloric acid, and measured by atomic fluorescence spectrometry. Se content was calculated as:

(2) $$\omega =\frac{(\rho -\rho 0)\times V}{m\times 1000}$$

where ω is Se content (mg/kg); ρ is Se concentration in the sample solution (μg/L); ρ0 is Se concentration in the blank (μg/L); V is final volume (mL); m is sample mass (g); 1000 is the unit conversion factor.

Organic selenium proportion was calculated as follows:

(3) $$Organic\;selenium\;proportion=\frac{\mathrm{Total}\;\mathrm{Se}-\mathrm{Inorganic}\;\mathrm{Se}}{\mathrm{Total}\;\mathrm{Se}}$$

where Total Se and Inorganic Se are the contents of total selenium and inorganic selenium in fruiting bodies, respectively (mg/kg).

#### 2.4.2. Selenium Speciation

After grinding, samples were extracted in water, hydrolyzed with protease, and analyzed by HPLC-ICP-MS for selenoamino acids and inorganic Se species, including methylselenocysteine (MeSeCys), selenocystine (SeCys2), selenomethionine (SeMet), selenite Se(IV), and selenate Se(VI). Component content was calculated as:

(4) $$W=\frac{(C-C_{0})\times V}{m\times 1000}$$

where W is component content (mg/kg); *C* is component concentration in the sample solution (μg/L); *C*_0_ is component concentration in the blank (μg/L); *V* is final volume (mL); *m* is the corresponding sample mass (g); 1000 is the conversion factor.

#### 2.4.3. Agronomic Traits

Fifty fruiting bodies were randomly selected per treatment. Pileus width and thickness were measured using a vernier caliper, and fresh weight per mushroom was measured using an electronic balance. Biological efficiency (BE) refers to the conversion efficiency of substrate into fruiting bodies and is an important indicator for evaluating the cultivation performance of edible fungi. BE (%) was calculated using the following formula:

(5) $$\mathrm{BE}\;\left(\%\right)=\frac{\mathrm{Wfr}}{Ws}\times 100$$

where Wfr is the total fresh weight of fruiting bodies (unit: grams) and Ws is the initial dry weight of the substrate (unit: grams).

#### 2.4.4. Nutritional Quality Indices

Crude protein was determined by the Kjeldahl method (GB 5009.5-2016) [26], crude fat by Soxhlet extraction (GB 5009.6-2016) [27], crude polysaccharides by the phenol–sulfuric acid method [28], and amino acid composition by an automatic amino acid analyzer (Hitachi L-8900, Tokyo, Japan; GB 5009.124-2016) [29].

### 2.5. Data Processing

Data were organized in Excel 2025 and analyzed using SPSS 27.0. One-way ANOVA was used to test differences among treatments, and Duncan’s multiple range test was applied for post hoc comparisons at *p* < 0.05. Figures were generated using Origin 2023. Data are presented as mean ± SD.

## 3. Results

### 3.1. Effects of Different Se Sources on Mycelial Growth

**Figure 1:**
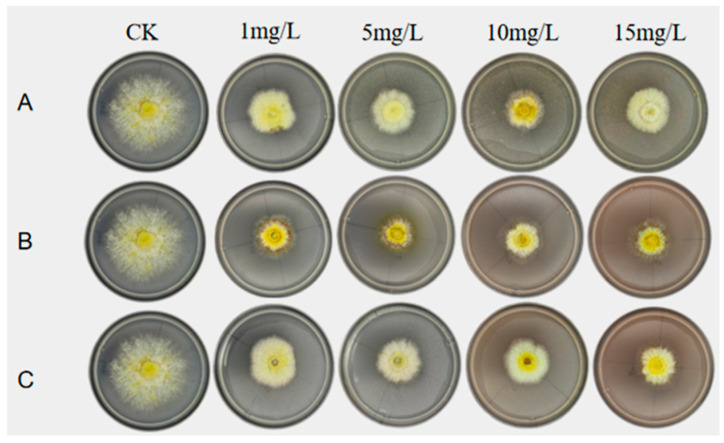
Colony morphology of *S. sanghuang* mycelia under different Se sources and concentrations. PDA plates supplemented with 1, 5, 10, and 15 mg/L Se. CK, no Se. (**A**) Se-yeast; (**B**) sodium selenite; (**C**) nano-Se.

To determine the suitability of different Se sources during the mycelial stage, PDA plate assays were performed with sodium selenite, nano-Se, and Se-yeast at 0–15 mg/L. Colony morphologies are shown in Figure 1. All exogenous Se treatments inhibited mycelial growth to varying degrees, manifested by weakened vigor and reduced colony density, with inhibition intensifying as concentration increased. At the same concentration, sodium selenite caused the strongest inhibition and produced sparse colonies; nano-Se showed intermediate effects; Se-yeast produced the best growth and the densest colonies.

Growth rate measurements and qualitative assessments (Table 1) corroborated these observations. Compared with CK, all Se treatments significantly reduced growth rate. At 5 and 10 mg/L, Se-yeast resulted in significantly higher growth rates than sodium selenite and nano-Se. Together with colony morphology, Se-yeast exhibited the most favorable overall mycelial performance, indicating higher physiological tolerance to organic Se than to inorganic Se or nano-Se, thereby providing a basis for Se source selection for fruiting cultivation.

### 3.2. Effects of Different Se Sources on Se Content in Mycelia

**Figure 2:**
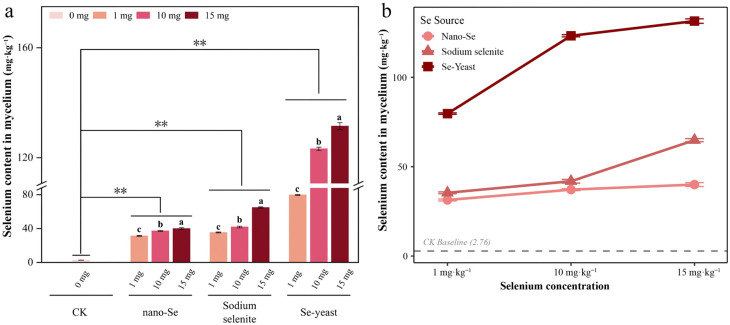
Effects of Se source and concentration on Se content in *S. sanghuang* mycelia. (**a**) Se content under different treatments; different lowercase letters indicate significant differences (*p* < 0.05). The symbol ** indicates a statistically highly significant difference (*p* < 0.01) between the control (no Se) and the respective Se treatment groups. (**b**) Interaction effect between Se source and concentration (two-way ANOVA); slope differences indicate a significant interaction (*p* < 0.001). Data are mean ± SD (*n* = 3).

Based on the growth inhibition differences, Se uptake and biotransformation efficiencies were further evaluated. As shown in Figure 2a, relative to CK, all three Se sources significantly increased Se content in mycelia, and the magnitude increased with concentration. Se-yeast showed the strongest enrichment effect: at 15 mg/L, mycelial Se content reached 131.47 mg/kg, which was 2.0-fold that of sodium selenite (64.86 mg/kg) and 3.3-fold that of nano-Se (40.02 mg/kg).

Two-way ANOVA (Figure 2b) revealed a significant interaction between Se source and concentration. Nano-Se displayed the flattest concentration–response slope, suggesting saturated or low-responsive uptake, whereas Se-yeast exhibited the steepest slope, indicating high sensitivity and strong uptake/biotransformation potential toward organic Se. Combined with the mycelial growth results, Se-yeast demonstrated dual advantages (minimal inhibition and maximal enrichment) and was therefore prioritized for subsequent fruiting-stage evaluation.

### 3.3. Effects of Different Se Sources on Fruiting Development and Agronomic Traits

**Figure 3:**
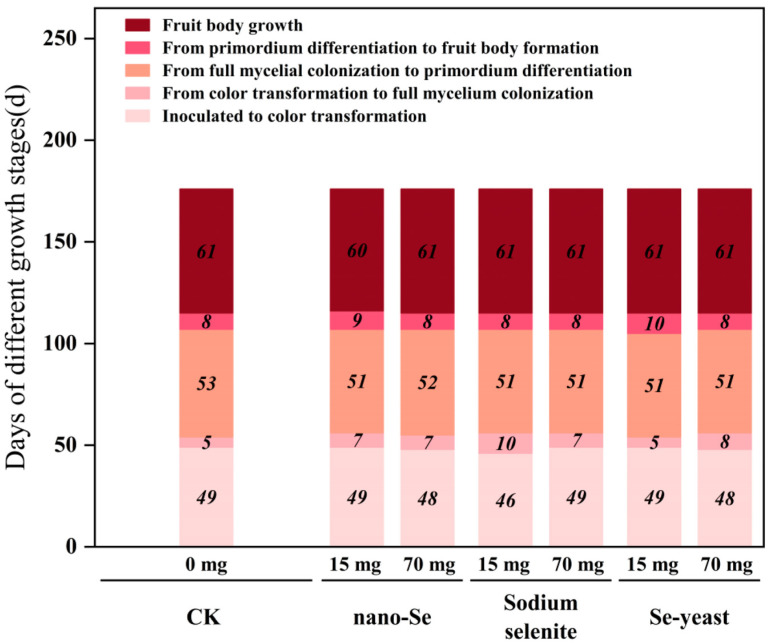
Effects of exogenous Se on the development of *S. sanghuang* fruiting bodies. Different colors represent different developmental stages; numbers indicate days required for each stage.

Following mycelial-stage screening, bag cultivation was conducted to assess the regulatory effects of different Se sources on fruiting body development and yield. The developmental timeline was shown in Figure 3. Regarding spawn run, most Se treatments delayed full colonization by 1–2 d compared with CK; notably, 15 mg/kg Se-yeast did not prolong colonization time, consistent with its weakest inhibitory effect observed on plates. In the color-transition stage, 15 mg/kg sodium selenite advanced coloration by 3 d relative to CK, whereas nano-Se and Se-yeast did not differ markedly from CK, suggesting that inorganic Se may accelerate physiological maturation via oxidative stress induction.

Post-harvest agronomic measurements (Table 2) demonstrated significant regulation by both Se source and concentration. At 15 mg/kg, Se-yeast produced significantly greater pileus width, fresh weight per mushroom, single-bag yield, and biological efficiency than sodium selenite and nano-Se at the same concentration; pileus thickness was second only to CK. These results indicated that an appropriate level of organic Se not only avoided suppressing fruiting development but can enhance yield through improved nutrient accumulation. In sharp contrast, 15 mg/kg nano-Se resulted in lower single-bag yield and biological efficiency than CK, indicating the most pronounced negative impact on fruiting at this dosage.

At 70 mg/kg, responses were more complex. Sodium selenite achieved the highest single-bag yield and biological efficiency among all treatments, but pileus thickness and fresh weight per mushroom were lower than those under 15 mg/kg Se-yeast, suggesting that high-yield performance under high-dose inorganic Se was mainly attributable to increased fruiting number rather than increased individual size, and was accompanied by physiological stress such as delayed colonization. From an industrial perspective, 15 mg/kg Se-yeast achieved high yield with a Se-source cost of only 0.36 RMB per bag, substantially lower than the high-dose sodium selenite treatment required to reach comparable yield. Overall, considering agronomic traits, yield performance, and economic efficiency, 15 mg/kg Se-yeast was identified as the optimal treatment and selected for further analyses.

### 3.4. Effects of Different Se Sources on Total Se in Fruiting Bodies

**Figure 4:**
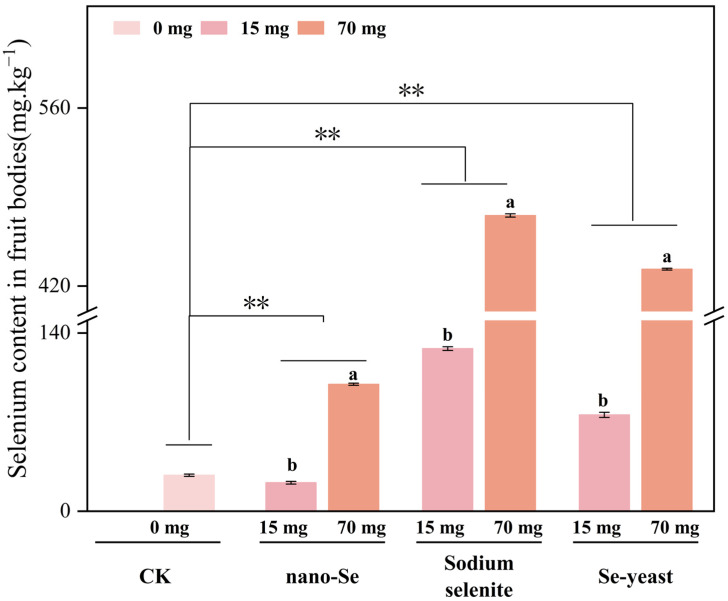
Se content in *S. sanghuang* fruiting bodies under different Se sources (sodium selenite, nano-Se, Se-yeast) and concentrations (15, 70 mg/kg). CK, no Se. Different lowercase letters indicate significant differences (*p* < 0.05). The symbol ** indicates a statistically highly significant difference (*p* < 0.01) between the control (no Se) and the respective Se treatment groups.

After defining the optimal treatment, Se accumulation in fruiting bodies under different Se sources and concentrations was compared (Figure 4). All treatments significantly increased fruiting-body Se relative to CK, but enrichment efficiency varied substantially among Se sources. At 15 mg/kg, sodium selenite yielded the highest Se content, slightly exceeding Se-yeast, while nano-Se was the lowest. This indicated that at low dosage, both inorganic and organic Se can effectively promote Se uptake in fruiting bodies with comparable efficiency, whereas nano-Se was markedly less effective.

At 70 mg/kg, Se contents increased sharply across treatments, and differences among sources became more pronounced. Sodium selenite reached the highest Se content (475.48 mg/kg), significantly higher than Se-yeast (433.30 mg/kg), while nano-Se remained low (98.76 mg/kg). This suggested that at high dosage, sodium selenite had a stronger accumulation advantage, potentially due to rapid transport via ionic channels and storage in fruiting tissues. Importantly, despite its higher Se accumulation, high-dose sodium selenite was accompanied by delayed mycelial growth and compromised morphology. In contrast, Se-yeast achieved high Se content while maintaining superior growth and yield performance.

### 3.5. Selenium Occurrence Characteristics in Fruiting Bodies Under the Optimal Treatment

**Figure 5:**
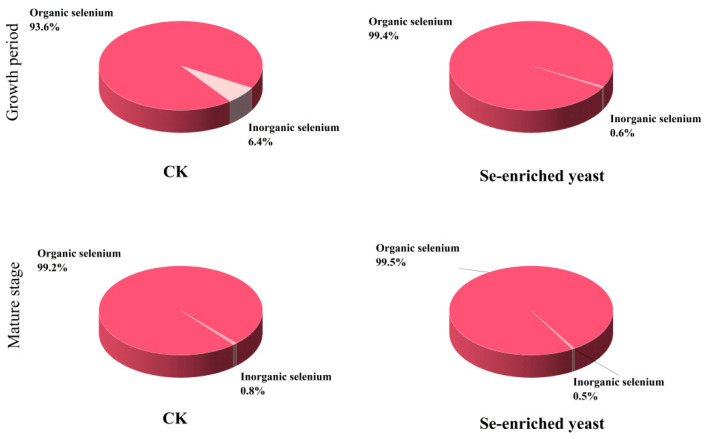
Proportions of organic and inorganic Se in *S. sanghuang* fruiting bodies at different developmental stages under Se-yeast treatment. CK, no Se; Se-enriched yeast, 15 mg/kg Se-yeast. Data are expressed as percentages of total Se.

After identifying 15 mg/kg Se-yeast as the optimal treatment, Se occurrence forms were further analyzed in the untreated control and this selected optimal treatment. Since 15 mg/kg Se-yeast showed the best overall performance in terms of agronomic traits, selenium enrichment, nutritional quality, and cost-effectiveness, selenium speciation analysis in this study was focused on these two groups. Figure 5 compared the proportions of organic and inorganic Se in CK and Se-yeast groups at the growth stage and mature stage. At both stages, Se-yeast treatment exhibited significantly higher organic Se proportions than CK. At the growth stage, CK contained 93.6% organic Se and 6.4% inorganic Se, whereas Se-yeast reached 99.4% organic Se with only 0.6% inorganic Se. This indicated that Se-yeast markedly promoted conversion of absorbed Se into organic forms and minimizes inorganic Se residues. From growth to maturity, organic Se proportions increased in both groups: CK rose from 93.6% to 99.2% (inorganic Se decreased from 6.4% to 0.8%), while Se-yeast increased from 99.4% to 99.5% (inorganic Se decreased from 0.6% to 0.5%). These findings suggested that the proportion of organic selenium increased naturally with development, but Se-yeast enabled near-complete conversion of selenium into organic forms already at the early growth stage. Notably, the organic Se proportion in Se-yeast at the growth stage exceeded that of CK at maturity, implying that Se-yeast not only improved the final organic Se fraction but also accelerated the biotransformation process.

**Figure 6:**
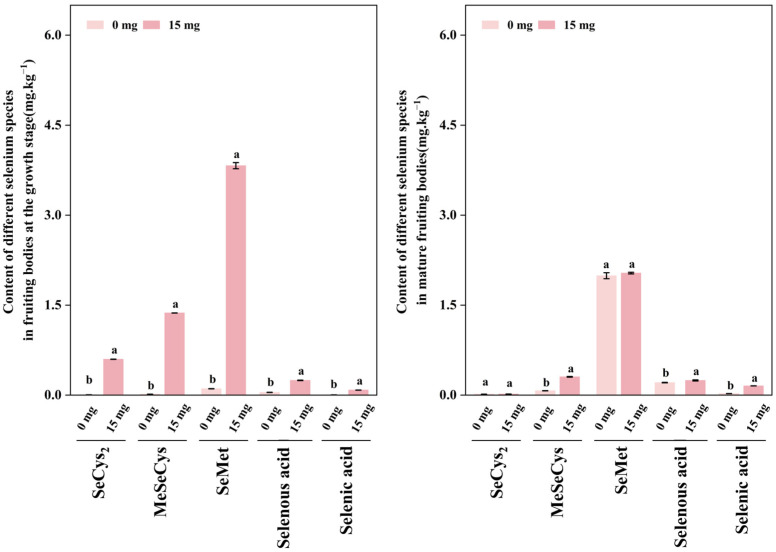
Se speciation profiles in *S. sanghuang* fruiting bodies under different treatments and developmental stages. CK vs. 15 mg/kg Se-yeast at growth and mature stages. Different lowercase letters indicate significant differences for the same Se species among treatments/stages (*p* < 0.05).

Figure 6 showed the contents of five Se species in fruiting bodies. Overall, SeMet was the predominant organic Se form, followed by MeSeCys and SeCys2, while inorganic Se levels were extremely low. Compared with CK, Se-yeast significantly increased all organic Se species. At the growth stage, SeMet and MeSeCys in the treatment group were substantially higher than those in CK at the same stage, indicating early high-level accumulation of organic Se under Se-yeast. Interestingly, Se species dynamics differed between groups from growth to maturity. In the Se-yeast group, all measured Se species decreased, whereas in CK they increased. This opposite trend led to a convergence of organic Se species levels between groups at maturity. This phenomenon may reflect distinct Se metabolic allocation strategies: under Se-yeast, organic Se rapidly accumulated early may be progressively incorporated into macromolecules (e.g., proteins) or shifted into other bound forms during maturation, thereby decreasing the detectable free species; in CK, organic Se may accumulate gradually throughout development, reaching higher levels only at maturity. These results indicated that Se-yeast not only increased total Se but also reshaped Se metabolic partitioning within fruiting bodies.

### 3.6. Effects of Se-Yeast on Nutritional Quality of Fruiting Bodies

**Figure 7:**
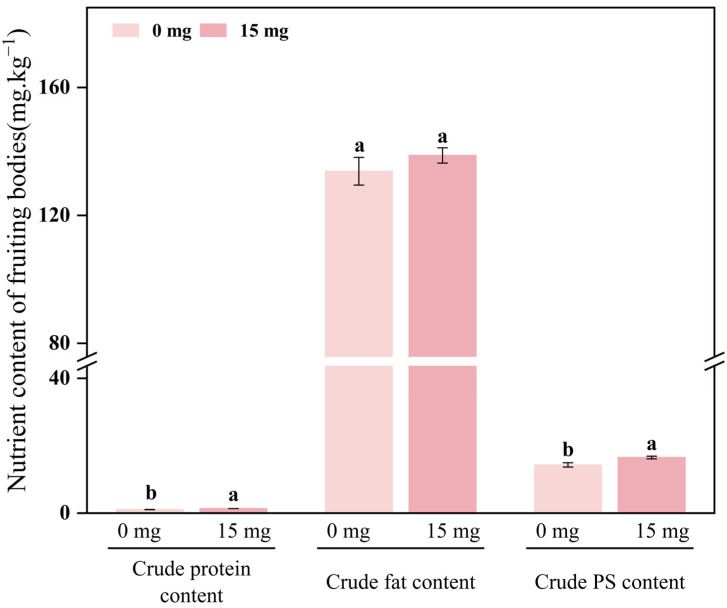
Effects of 15 mg/kg Se-yeast on crude protein, crude fat, and crude polysaccharides in *S. sanghuang* fruiting bodies. Different lowercase letters indicate significant differences within each component (*p* < 0.05).

Given that 15 mg/kg Se-yeast combined favorable agronomic traits, high Se enrichment, and high organic conversion, its effects on nutritional quality were further assessed. As shown in Figure 7, Se-yeast significantly increased major nutritional components in fruiting bodies. Crude protein, crude fat, and crude polysaccharides were all higher than in CK, indicating that an appropriate level of organic Se promotes accumulation of fundamental nutrients.

**Figure 8:**
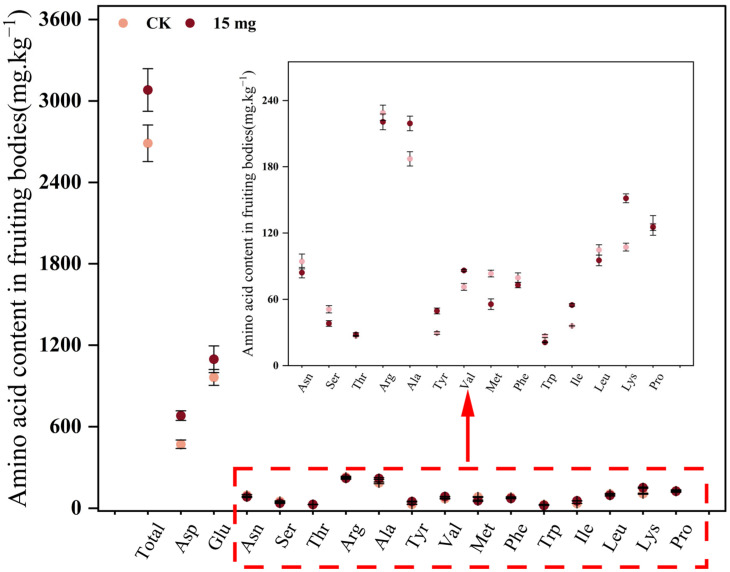
Effects of 15 mg/kg Se-yeast on amino acid composition and contents in *S. sanghuang* fruiting bodies.

Amino acid analysis (Figure 8) showed that total amino acids increased by 14.63% under 15 mg/kg Se-yeast compared with CK. Se-yeast selectively promoted the synthesis of aspartic acid, glutamic acid, and lysine, with lysine exhibiting the largest increase (41.15%). In contrast, methionine and tryptophan were relatively higher in CK. This selective regulation suggested that Se exerted fine-tuned control over amino acid metabolism in *S. sanghuang*, improving certain flavor-related amino acids and essential amino acids, thereby providing a strong quality foundation for high-value Se-enriched *Sanghuang* products.

## 4. Discussion

Edible fungi can serve as efficient biotransformation systems for selenium, converting inorganic Se into organic forms with higher bioavailability. Recent studies have further highlighted the potential of selenium-enriched edible and medicinal mushrooms as functional foods because selenium fortification can simultaneously improve selenium nutrition, regulate selenium speciation, and enhance product quality [10,11,30]. However, most previous studies have focused on common cultivated mushrooms, while selenium-enrichment strategies for high-value medicinal fungi remain relatively limited [11,13,30]. This study systematically compared sodium selenite, nano-Se, and Se-yeast with respect to mycelial growth, fruiting development, Se enrichment/speciation, and nutritional quality in *S. sanghuang*, demonstrating pronounced Se-source specificity and concentration dependence.

At the mycelial stage, all Se sources inhibited growth in a concentration-dependent manner, with sodium selenite showing the strongest inhibition and Se-yeast the weakest. Meanwhile, Se-yeast achieved the highest mycelial Se content (131.47 mg/kg), 2.0-fold and 3.3-fold higher than sodium selenite and nano-Se, respectively. These patterns were consistent with previous findings that mycelial inhibition intensified with increasing Se concentration and that inorganic Se generally exerted greater toxicity than organic Se [30,31,32,33,34,35]. Selenium tolerance thresholds differed across mushroom species [11,13,30,31,34,35]; here, *S. sanghuang* was relatively sensitive to sodium selenite, showing marked inhibition at 15 mg/L. Therefore, the responses observed in *S. sanghuang* should be interpreted in a species-specific context rather than directly compared with those of other selenium-enriched mushrooms. Two-way ANOVA confirmed a significant Se-source × concentration interaction, and the steepest enrichment response under Se-yeast suggested exceptionally strong uptake/biotransformation capacity for organic Se.

During fruiting, 15 mg/kg Se-yeast was optimal: it did not prolong full colonization time and significantly improved pileus width, fresh weight per mushroom, single-bag yield, and biological efficiency versus other treatments. Sodium selenite at 15 mg/kg advanced coloration by 3 d but did not translate into yield advantage, while nano-Se consistently produced the poorest agronomic outcomes. These results align with studies in *Pleurotus ostreatus* [16] and *Auricularia* spp. [31], indicating that appropriate Se levels can increase yield, whereas unsuitable sources or excessive dosages can induce stress [36]. Notably, high-dose sodium selenite (70 mg/kg) produced the highest fruiting-body Se (475.48 mg/kg) and yield but was associated with delayed mycelial growth and inferior morphology, suggesting that excessive accumulation comes at a physiological cost. Therefore, for practical cultivation of *S. sanghuang*, the optimal selenium-fortification strategy should not be defined solely by maximal selenium accumulation but rather by integrated performance including growth stability, yield, selenium speciation, nutritional quality, and economic feasibility.

Selenium bioefficacy depends strongly on chemical speciation. Under 15 mg/kg Se-yeast, the organic Se proportion reached 99.4% already at the growth stage—exceeding the mature-stage level of CK (99.2%)—and further increased to 99.5% at maturity, indicating that Se-yeast enhanced total Se while accelerating the conversion of selenium into organic forms. Speciation analysis showed that SeMet and MeSeCys were dominant, consistent with Se-enriched *Lentinula edodes* [37] and *Cordyceps militaris* [38]. The opposing developmental trends in detectable Se species between Se-yeast and CK suggest that Se-yeast alters Se allocation, potentially shifting early accumulated free organic Se into protein-bound or other conjugated forms during maturation. It should be noted that selenium speciation in the present study was analyzed only for the control and the selected optimal treatment (15 mg/kg Se-yeast), which does not allow a direct comparison of selenium species among sodium selenite, nano-Se, and Se-yeast treatments. Previous studies have shown that selenium source can markedly affect selenium accumulation, speciation, and bioaccessibility in edible mushrooms [11,14,16,36,38]. Therefore, selenium accumulation and selenium speciation should be considered separately when evaluating selenium-enriched mushrooms. In particular, higher total selenium content under inorganic selenium treatments may not necessarily correspond to proportionally higher incorporation into organic selenium forms [14,16,17,36]. Future studies should further compare selenium speciation among different selenium sources to clarify the relationship between selenium accumulation and selenium biotransformation in *S. sanghuang*.

In addition, the significance of the present study should also be considered in relation to the target species. Unlike common edible mushrooms, *S. sanghuang* is a high-value medicinal fungus with recognized bioactive potential. Recent studies on Sanghuangporus species have highlighted their rich bioactive composition and antioxidant-related activities, supporting their importance as medicinal fungal resources [3]. Therefore, selenium biofortification in *S. sanghuang* is meaningful not only for improving selenium nutrition but also for developing value-added functional products based on medicinal mushrooms. In this sense, the importance of our findings lies not simply in achieving the highest selenium accumulation but in establishing a practical selenium-enrichment strategy for a medicinal mushroom with excellent selenium organicization and acceptable agronomic performance.

Selenium has been reported to improve nutritional quality and bioactivity in edible fungi [39]. Here, 15 mg/kg Se-yeast significantly increased crude protein, crude polysaccharides, and total amino acids, with lysine showing the greatest increase. Given lysine’s importance as a limiting essential amino acid, this enhancement supports functional development of Se-enriched *Sanghuang*. In addition, the detection of MeSeCys together with the extremely high proportion of organic selenium (>99.5%) further suggests the potential functional value of Se-yeast-enriched *S. sanghuang* [30,37,38]. Economically, the Se-source cost for 15 mg/kg Se-yeast was only 0.36 RMB per bag, delivering favorable cost-effectiveness relative to sodium selenite and nano-Se.

In summary, 15 mg/kg Se-yeast achieved high yield, a very high organic selenium proportion, and improved nutritional quality, representing the best overall option for Se-enriched Sanghuang cultivation. The value of this study lies not only in selenium enrichment itself but also in providing a practical and economically feasible strategy for standardized cultivation of selenium-enriched *S. sanghuang* as a medicinal mushroom product. Future work should further resolve the distribution of bound Se pools and their bioactivities.

## 5. Conclusions

By systematically comparing sodium selenite, nano-Se, and Se-yeast, this study demonstrated that *S. sanghuang* exhibits pronounced Se-source-specific and concentration-dependent responses. Se-yeast caused the least inhibition of mycelial growth while achieving the highest Se uptake and biotransformation efficiency; in bag cultivation, 15 mg/kg Se-yeast delivered the best overall performance by significantly increasing pileus width, fresh weight per fruiting body, single-bag yield, and biological efficiency without extending full colonization time and with superior cost-effectiveness. This treatment markedly promoted the conversion of selenium into organic forms, yielding an extremely high organic Se proportion (>99.5%) dominated by selenomethionine, and showed an opposite developmental trend of detectable Se species relative to the control, suggesting altered Se metabolic partitioning. In addition, 15 mg/kg Se-yeast significantly enhanced crude protein, crude polysaccharides, and total amino acids, with lysine exhibiting the greatest increase. Collectively, Se-yeast is the optimal Se source for Se-enriched *Sanghuang*, with 15 mg/kg recommended for standardized production and functional product development. However, because selenium speciation was characterized only for the selected optimal treatment in this study, future work should further compare selenium speciation among inorganic, nano-, and organic selenium sources to better elucidate the relationship between selenium accumulation and selenium biotransformation in *S. sanghuang*.

## Figures and Tables

**Table 1:** Mycelial growth rate and qualitative assessment under different Se sources and concentrations on PDA. CK, no Se. Values are mean ± SD. Different lowercase letters indicate significant differences (*p* < 0.05). ++++/+++/++ denote strong/moderate/weak growth. “Growth status” describes color and density.

Selenium Sources and Concentration (mg/L)	Growth Rate (mm/d)	Mycelium Growth Assessment	Mycelium Growth Status
CK	2.53 ± 0.18 ^a^	++++	Bright yellow, dense
Sodium selenite (1)	1.811 ± 0.01 ^b^	++	Bright yellow, sparse
Sodium selenite (5)	1.651 ± 0.03 ^c^	++	Bright yellow, sparse
Sodium selenite (10)	1.511 ± 0.08 ^c^	++	Bright yellow, sparse
Sodium selenite (15)	1.781 ± 0.02 ^b^	++	Pale yellow, sparse
nano-Se (1)	2.071 ± 0.03 ^bc^	+++	Bright yellow, dense
nano-Se (5)	1.86 1 ± 0.01 ^c^	+++	Bright yellow, relatively dense
nano-Se (10)	1.64 ± 0.01 ^bc^	+++	Bright yellow, relatively dense
nano-Se (15)	1.63 ± 0.09 ^bc^	+++	Pale yellow, relatively dense
Se-yeast (1)	1.91 ± 0.01 ^b^	+++	Pale yellow, relatively dense
Se-yeast (5)	1.90 ± 0.04 ^b^	+++	Pale yellow, relatively dense
Se-yeast (10)	1.70 ± 0.03 ^bc^	++	Bright yellow, sparse
Se-yeast (15)	1.59 ± 0.02 ^c^	+++	Pale yellow, relatively dense

**Table 2:** Agronomic traits of *S. sanghuang* fruiting bodies under different Se sources and concentrations in substrate. CK, no Se. Values are mean ± SD. Different lowercase letters indicate significant differences (*p* < 0.05).

Selenium Sources and Concentration (mg/kg)	Pileus Width (mm)	Pileus Thickness (mm)	Fresh Weight of Single Mushroom (g)	Single Bag Yield (g)	Biological Efficiency (%)	Selenium Source Price (RMB, CNY)
CK	87.35 ± 2.78 ^a^	23.82 ± 0.81 ^a^	35.16 ± 5.94 ^a^	46.88 ± 2.81 ^c^	9.38 ± 2.72 ^c^	
Sodium selenite (15)	84.68 ± 0.28 ^a^	18.64 ± 1.51 ^c^	33.77 ± 0.19 ^b^	67.54 ± 3.34 ^b^	13.51 ± 0.19 ^b^	0.41
Sodium selenite (70)	84.99 ± 5.51 ^b^	20 ± 0.12 ^b^	32.52 ± 3.11 ^b^	71.55 ± 6.25 ^a^	14.31 ± 3.11 ^a^	1.93
nano-Se (15)	76.39 ± 3.89 ^c^	19.17 ± 0.54 ^b^	30.22 ± 1.22 ^c^	36.16 ± 0.75 ^d^	7.22 ± 1.22 ^d^	0.16
nano-Se (70)	82.47 ± 0.83 ^bc^	16.91 ± 1.84 ^c^	26.49 ± 0.24 ^d^	46.99 ± 1.01 ^c^	9.4 ± 0.24 ^c^	0.77
Se-yeast (15)	88.97 ± 0.51 ^b^	21.58 ± 0.12 ^b^	33.75 ± 2.27 ^b^	67.50 ± 6.90 ^a^	13.5 ± 2.37 ^a^	0.36
Se-yeast (70)	74.15 ± 1.21 ^c^	18.44 ± 0.49 ^bc^	25.48 ± 0.88 ^d^	50.95 ± 5.50 ^c^	10.19 ± 0.88 ^c^	1.68

## Data Availability

The original contributions presented in the study are included in the article, further inquiries can be directed to the corresponding authors.
